# Supplementary material for: Serum wisteria floribunda agglutinin-positive human Mac-2 binding protein is unsuitable as a diagnostic marker of occult hepatocellular carcinoma in end-stage liver cirrhosis
Source: PLoS One. 2023 Nov 1;18(11):e0293593. doi: 10.1371/journal.pone.0293593 (PMC10619783; doi:10.1371/journal.pone.0293593)
Supplement: S1 File — (DOCX) [file pone.0293593.s001.docx]

Reference data

|  | N group | D group | I group | DS group |
| --- | --- | --- | --- | --- |
| Case number | 30 | 10 | 41 | 32 |
| Age | 57 (26 - 68) | 54 (33 - 69) | 60 (48 - 71) | 60.5 (52 – 72) |
| Sex (M : F) | 15 : 15 | 5 : 5 | 31 : 10 | 19 : 13 |
| HBV | 2 | 2 | 7 | 5 |
| HCV | 6 | 6 | 27 | 14 |
| Co-infection HBV/HCV | 0 | 1 | 2 | 0 |
| non B, non C | 22 | 1 | 5 | 13 |
| Child-Pugh score | 11 (7 - 14) | 9 (6 – 14) | 10 (5 – 13) | 10 (7 – 13) |
| MELD score | 21 (8 – 39) | 13 (7 – 29) | 13 (4 – 31) | 14 (7 – 26) |
| M2BP index | 9.125 (1.25–18.79) | 11.03 (1.01–18.21) | 9.67 (0.29–17.83) | 9.56 (0.28–19.44) |

| Comparison between the presence or absence of histological HCC and M2BPGi | | | | | |
| --- | --- | --- | --- | --- | --- |
|  | the presence of HCC | | the absence of HCC | | p value |
| M2BP index | 9.59 (0.28 - 19.44) | | 9.125 (1.25–18.79) | | 0.573 |
| The ROC curve analysis for the diagnosis of HCC including occult HCC | | | | | |
|  | Cut-off value | AUC | Sensitivity | Specificity | p-value |
| M2BP index | 7.98 | 0.5347 | 0.4096 | 0.7666 | 0.543 |

We compared between N, D, I, DS group after increasing N group to 30 patients. Whole livers of these patients were diagnosed pathologically as non HCC. But these pathological tests weren’t done with the protocol used in this study. Including this data, there was no differences in M2BP between their groups.
