# Supplementary material for: Serum wisteria floribunda agglutinin-positive human Mac-2 binding protein is unsuitable as a diagnostic marker of occult hepatocellular carcinoma in end-stage liver cirrhosis
Source: PLoS One. 2023 Nov 1;18(11):e0293593. doi: 10.1371/journal.pone.0293593 (PMC10619783; doi:10.1371/journal.pone.0293593)
Supplement: Attachment — Submitted filename: Response to Reviewers.docx [file pone.0293593.s003.docx]

**Answers to review comments**

Review Comments to the Author

Please use the space provided to explain your answers to the questions above. You may also include additional comments for the author, including concerns about dual publication, research ethics, or publication ethics. (Please upload your review as an attachment if it exceeds 20,000 characters)

Reviewer #1: The authors addressed the reviewers' comments properly. Although the sample size is relatively small, the study design that assesses the whole explanted liver is highly original. The reviewer thinks that the paper has a clinical impact on understanding the utility of M2BP.

→ **Thank you for your kind comments. You pointed out exactly our strength and weakness. However, we believe that our strength of whole liver histological examination is unique and impactful.**

Reviewer #2: It seems to have been improved with revision. Stratified by the background hepatic disease, the number of each background is too small. Therefore, it is difficult to judge whether it is correct.

→ **Thank you for your pointing it out. We understand the small number of them as referred to in limitation. We would like to keep further study.**

Reviewer #3: Nagakawa K et al. demonstrated that serum WFA+-M2BP level was not associated with the presence of occult HCC in the explanted liver in liver transplantation. The author appropriately revised the manuscript according to the reviewer’s comments, and the manuscript is suitable for publication in PLOS one in its present form.

→ **Thank you for your kind comments. I’m glad that you accepted our revises.**

Reviewer #4: Thank you very much for the opportunity to review this manuscript. I have no further comment for this paper.

→ **Thank you very much too. I’m really happy that you accepted our revision.**

Reviewer #5: Authors have revised the manuscript. And I confirmed this manuscript has partially improved. However, there is an important point to be clarified. It is still difficult to understand the impact and utility of the negative clinical data regarding M2BPGi. The authors should emphasize the significance and practical implications of this study for clinical practice in the discussion section. Could you please explain why the authors emphasize M2BPGi in the title (including the short title)?"

→ **Thank you for your important query. In actually, there were many papers regarding that M2BPGi is a predicting marker of HCC, as mentioned in Discussion. Moreover, Chuaypen et al. literally said M2BPGi represented a sensitive marker of early stage HCC. So, it is reasonable for us to emphasize whether M2BPGi could be a diagnostic marker of occult HCC. We added the below sentences in Discussion to tell why we emphasized M2BPGi in the title.**

**Line 188-191; In the study, the authors concluded serum WFA+-M2BP represented a sensitive marker of early-stage HCC detection in patients with chronic HBV infection. [4] This conclusion motivated us to investigate WFA+-M2BP as a detecting and diagnostic marker of occult HCC.**

[4] Chuaypen N, Chittmittraprap S, Pinjaroen N, Sirichindakul B, Poovorawan Y, Tanaka Y, et al. Serum Wisteria floribunda agglutinin-positive Mac-2 binding protein level as a diagnostic marker of hepatitis B virus-related hepatocellular carcinoma. Hepatol. Res. 2018;48:872–881.
